# Overactive bladder in an integrated delivery system: a longitudinal cohort study

**DOI:** 10.1186/s12913-020-05315-1

**Published:** 2020-05-20

**Authors:** Jeffrey A. Linder, Joel S. Weissman, Harry Reyes Nieva, Stuart Lipsitz, R. Sterling Haring, Julie DeAngelis, Rita M. Kristy, Kevin R. Loughlin

**Affiliations:** 1 Division of General Internal Medicine and Geriatrics, Northwestern University Feinberg School of Medicine, 750 N. Lake Shore Drive, 10th Floor, Chicago, IL 60611 USA; 2 Center for Surgery and Public Health, Brigham and Women’s Hospital, Harvard Medical School, Boston, MA USA; 3 Division of General Internal Medicine and Primary Care, Brigham and Women’s Hospital, Harvard Medical School, Boston, MA USA; 4 Department of Physical Medicine and Rehabilitation, Vanderbilt University Medical Center, Nashville, TN USA; 5 Astellas Pharma Global Development, Northbrook, IL USA; 6 Division of Urology, Brigham and Women’s Hospital, Harvard Medical School, Boston, MA USA

**Keywords:** Antimuscarinics, Anticholinergic burden, Electronic health records, Integrated delivery system, Medication and procedure use, Overactive bladder, Overactive bladder syndrome, Primary and specialty care, Urgency, Urinary incontinence

## Abstract

**Background:**

Overactive bladder (OAB) is common and morbid. Medication and diagnosis claims may be specific, but lack sensitivity to identify patients with overactive bladder. We used an “electronic health record (EHR) phenotype” to identify cases and describe treatment choices and anticholinergic burden for OAB.

**Methods:**

We conducted a retrospective cohort study in a large, integrated health delivery system between July 2011 and June 2012 (2-year follow-up). We examined care from primary care and specialty clinics, medication and procedure use, and anticholinergic burden for each patient.

**Results:**

There were 7362 patients with an EHR OAB phenotype; 50% of patients were > 65 years old, 74% were female, and 83% were white. The distribution of care included primary care physician (PCP)/specialty co-management (25% of patients); PCP care only (18%); urology only (13%); or some other combination of specialty care (33%). Only 40% of patients were prescribed at least 1 OAB medication during the study. The mean duration of prescribed medication was 1.5 months (95% confidence interval [CI], 1.4 to 1.6 months; range, < 1 month to 24 months). Independent predictors of receipt of an OAB medication included increasing age (odds ratio [OR], 1.4 for every 10 years; 95% CI, 1.4 to 1.5), women (OR, 1.6 compared with men; 95% CI, 1.4 to 1.8), diabetes (OR, 1.3; 95% CI, 1.1 to 1.5), and certain sources of care compared with PCP-only care: PCP/specialty co-management (OR, 1.8; 95% CI, 1.5 to 2.0), urology (OR, 2.2; 95% CI, 1.8 to 2.6), and multiple specialists (OR, 1.4; 95% CI, 1.2 to 1.8). Very few patients received other treatments: biofeedback (< 1%), onabotulinumtoxinA (2%), or sacral nerve stimulation (1%). Patients who received OAB medications had significantly higher anticholinergic burden than patients who did not (anticholinergic total standardized daily dose, 125 versus 46; *P* < .001).

**Conclusions:**

Although OAB is common and morbid, in a longitudinal study using an EHR OAB phenotype 40% of patients were treated with OAB medication and only briefly.

## Background

Overactive bladder (OAB) is defined by the International Continence Society (ICS) as a symptom syndrome defined by urinary urgency, frequency, and nocturia, with or without incontinence [1–3]. OAB is common and its prevalence can vary widely depending on the patient population and disease definition, affecting 7–27% of men and 9–43% of women [1, 2]. OAB is associated with impaired sleep quality, depression, falls, fractures, social isolation, and worse quality of life [1, 3].

OAB can be difficult-to-treat in part because of the anticholinergic side effects of the most commonly prescribed medications. Recently, anticholinergic medications, including OAB medications, have been implicated in the development of cognitive impairment and dementia [4, 5].

OAB is underreported by patients and poorly recognized in primary care [6]. OAB may also be under-recognized by specialists in integrated health systems. Prior studies have been limited by use of claims or prescribing data to identify OAB patients, which may focus only on highly-specified patient populations who have multiple visits for OAB, an explicit diagnosis of OAB, or OAB medication prescriptions [7–9]. Such methods may misclassify patients who present with the complex of symptoms that define OAB. In addition, prior studies have not described care of patients with OAB within an integrated delivery system utilizing electronic health records (EHRs), in which patients could receive care from some combination of primary and specialty care, including urology, gynecology, and urogynecology.

EHR-based disease phenotypes have the potential to more sensitively identify patient cohorts [10, 11]. Based on an initial chart review, modification, and refinement, EHR phenotypes use a mix of coded data and free-text criteria to identify patients with target conditions. EHR phenotypes are particularly useful for conditions like OAB that do not have reliable, well-defined diagnostic criteria (e.g., simply using International Statistical Classification of Diseases and Related Health Problems-9 [ICD-9] codes for OAB).

The aim of this study was to identify patients with an EHR OAB phenotype and conduct a health system-based, longitudinal cohort assessment to describe care by different specialties, medication use, and procedures, as well as to assess the anticholinergic burden of patients who did and did not receive OAB medications.

## Methods

### Setting, data source, and overview

Partners HealthCare is an integrated health delivery system that provides primary and specialty care in Eastern Massachusetts. The system has eight acute care hospitals including Brigham and Women’s Hospital and Massachusetts General Hospital. Partners HealthCare includes approximately 1000 primary care physicians (PCPs) and about 5500 specialists. All Partners HealthCare entities share a common data infrastructure.

**Fig. 1:**
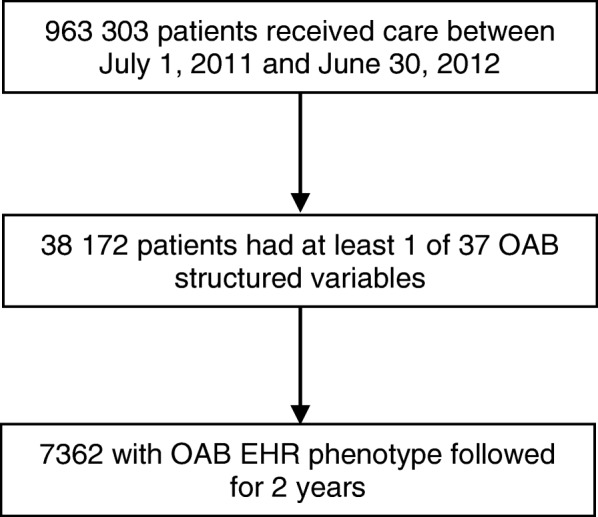
Patient Flow. OAB, overactive bladder; EHR, electronic health record

Because of limitations of prior methods of identifying patients with OAB, we developed an OAB EHR phenotype through identification of coded fields, a pilot chart review, a definitive chart review, and EHR model training. We retrospectively identified Partners HealthCare patients using an OAB EHR phenotype – a combination of structured variables and natural language processing of free text – who met criteria for OAB between July 1, 2011 and June 30, 2012 [10, 11]. We followed patients from their index visit – the date on which they met criteria for OAB – for 2 years. We examined visit, medication, and utilization data (Fig. 1).

### EHR OAB phenotype

#### Identification of OAB-related coded fields

The development of the OAB EHR phenotype began with two clinician investigators (JAL, KRL), with input from the rest of the study team, identifying 37 ICD-9 diagnosis codes, medications, or problem list entries that could be potentially associated with OAB (Supplementary Table 1). ICD-9 codes included “hypertonicity of bladder,” “retention of urine,” “urgency of urination,” and others. Problem list entries included “bladder dysfunction,” “hyperactive bladder,” “urgent desire to urinate,” and others. We designated each of the 37 variables as either “highly likely” to be associated with a patient who had a diagnosis of OAB or “likely” to be associated with a diagnosis of OAB.

#### Pilot chart review

To determine the most efficient sample size for a larger chart review, JAL and KRL conducted a pilot chart review of 20 charts with a highly likely code, 20 charts with only a likely code, and 20 charts with no OAB-related code. On chart review, highly likely codes had a positive predictive value of 70%, likely codes had a positive predictive value of 35%, and, for charts with no OAB-related code a PPV of 0%. Based on these estimates for the PPV of highly-likely and likely codes and the distribution of highly-likely and likely codes, we determined that, a sample size of 729 reviewed charts would yield a 95% CI (i.e., the 95% CI for the PPV would be at most 10% wide). We subsequently identified patients 18 years or older who had any of these 37 diagnosis codes, medications, or problem list entries.

#### Definitive chart review

Three physician reviewers examined a random sample of 729 charts to identify those which met pre-specified, database collected and calculated criteria, consistent with ICS and International Consultation on Incontinence definitions, for a clinical diagnosis of OAB [12]. For the chart review, we created four phenotype classifications (*definite*, *probable*, *possible, and not OAB*) based on a combination of features including the results of urodynamic testing or, in the absence of urodynamic testing, documentation in patient notes. Patients were considered to have *definite* OAB if they had urodynamic testing results of detrusor hyperreflexia, detrusor instability, uninhibited contraction, or equivalent language. For the vast majority of patients who did not have urodynamic testing, we required chart documentation of 3 symptoms: 1) ***urinary urgency*** in the absence of urinary tract infection, history of genitourinary procedure within the past 90 days, or other obvious pathology, 2) ***urinary frequency*** (voiding eight or more times or an unknown quantity per day), and 3) ***nocturia*** (awakening two or more times or an unknown number to void). We defined urgency as a sudden, compelling need to void which is difficult to defer. A patient was classified as having *definite* OAB if they had urgency, frequency, and nocturia; *probable* OAB if they had urgency and frequency; *possible* OAB if they had only urgency; and *not* OAB if they had none of the symptoms or had an alternative cause of symptoms. If a urinalysis showed five or more white blood cells per high power field, we required a negative culture to rule out alternate cause of urgency.

Reviewers also responded to the prompt, “Do you agree or disagree with the assigned category of the OAB diagnosis?” using a 4-point Likert scale (*completely agree*, *partially agree*, *partially disagree*, *completely disagree*). We considered OAB cases as those which were classified as definite OAB with which the reviewer completely agreed; definitely OAB with which the reviewer partially agreed; or classified as probable OAB with which the reviewer completely agreed.

#### OAB EHR phenotype based on coded fields and free-text

To develop our OAB EHR phenotype, we randomly selected 70% of patients classified by chart review for training and held the remaining 30% for the testing set. We trained an L1-regularized (Least Absolute Shrinkage and Selector Operator, LASSO) [13, 14] logistic regression using the gold standard labels assigned from chart review (1 for OAB; 0 otherwise) as the dependent variable and the coded and free-text elements in the EHR as model features. We incorporated free-text from outpatient clinical notes into our phenotyping algorithm via natural language processing. We aggregated all outpatient clinical notes into a single document for each patient and employed a “bag-of-words” (BOW) representation.

As determined a priori*,* we set the predictive probability of the LASSO model to maximize the sum of sensitivity and specificity (NLP-only model: sensitivity, 48%; specificity, 84%; c-statistic, 0.64; NPV, 89%; PPV, 37%). For the final predictive model, because we intended to examine medications as an outcome, we omitted medications as a coded field, sacrificing some PPV. The final model (Supplementary Table 2) had a sensitivity of 54%, specificity of 80%, c-statistic of 0.72, negative predictive value of 83%, and, consistent with our pilot and final chart review, a PPV of 54% (see Discussion).

### Data extraction and analysis

For cases that met the EHR phenotype definition of OAB, we extracted sociodemographic information including age, sex, race/ethnicity, primary language, insurance type, and marital status. Sociodemographic information was collected during patient registration at Partners HealthCare sites. In addition, we used the EHR problem list and diagnosis entries to determine if the patient had moderate-to-severe chronic kidney disease; chronic obstructive pulmonary disease; congestive heart failure; or diabetes mellitus (Supplementary Table 3). We also examined two relative contraindications to anticholinergic treatment: glaucoma and dementia.

We classified patients according to what specialties cared for the patient (source of care) over the following 2 years. We classified patients’ source of care as PCP only; a single “OAB-related specialist,” including urology, gynecology, or urogynecology; a combination of OAB-related specialists; co-management by a PCP and any OAB-related specialists; or other (ie, patients who did not receive care from a PCP, urologist, gynecologist, or urogynecologist).

We identified the first OAB-related medication that patients received during follow-up, including darifenacin, fesoterodine, flavoxate, hyoscyamine, imipramine, mirabegron, oxybutynin, solifenacin, tolterodine, and trospium. Among patients who received an OAB medication, we measured the number of different medications prescribed to individual patients over 2 years of follow-up. In addition, we measured the total number of months of medication each patient was prescribed and calculated the average duration of medication prescription coverage among patients who received any medication. We used Current Procedural Terminology (CPT) codes to identify procedures including biofeedback, onabotulinumtoxinA injections, and sacral nerve stimulation (Supplementary Table 4).

We measured anticholinergic burden of OAB and non-OAB medications using the method of Gray and colleagues [5, 15]. Briefly, we multiplied the medication strength by the number of units of medication dispensed to calculate a total medication dose for each anticholinergic medication, the anticholinergic total standardized daily dose (TSDD). We then converted the total dose for each medication into a standardized daily dose by dividing by the minimum reported effective dose of each medication. We then added the standardized daily dose across medications to calculate a TSDD for anticholinergic medications and examined the difference in anticholinergic TSDD between those who did and did not receive OAB medications.

### Statistical analysis

We described basic associations using proportions. We compared proportions – including sociodemographic, clinical, and treatment difference between men and women – using the chi-squared test. To examine independent predictors of receipt of OAB medications, we used multivariable logistic regression. We modeled age in decades and omitted insurance status, which was strongly colinear with age. Language was also omitted, as it was strongly colinear with race/ethnicity. We used the linear trend test to assess the relationship between number of OAB medications prescribed during the study period and increasing TSDD. For all analyses we used SAS (version 9.4, Cary, NC) and considered *P* values < .05 significant.

## Results

### Cohort derivation and characteristics

Of the 963,303 patients with EHR records during the study period, 38,172 had at least one coded variable that was potentially related to OAB. Of these, 7362 patients had an OAB EHR phenotype without medications as predictors. Of these patients, 50% were over 65 years old, 74% were female, and 83% were white (Table 1). In total, 45% of patients had private insurance, 47% had Medicare, and 5% had Medicaid. Comorbid conditions included chronic obstructive pulmonary disease (20%), diabetes (17%), congestive heart failure (9%), chronic kidney disease (4%), glaucoma (3%), and dementia (3%).

**Table 1:** Cohort Characteristics by Sex

Characteristic	Overall (*n* = 7362)	Female (*n* = 5417)	Male (*n* = 1945)	*P* value
	No. (column %)			
Less than 65 years old	3646 (50)	2797 (52)	849 (44)	< .001
65 years old or older	3716 (50)	2620 (48)	1096 (56)	
Total	7362 (100)	5417 (100)	1945 (100)	
Race/ethnicity				
White/Caucasian	6103 (83)	4516 (83)	1587 (82)	.012
Black/African American	338 (5)	233 (4)	105 (5)	
Hispanic/Latino	270 (4)	211 (4)	59 (3)	
Asian/Pacific Islander	125 (2)	82 (2)	43 (2)	
Other/unknown	526 (7)	375 (7)	151 (8)	
Total	7362 (100)	5417 (100)	1945 (100)	
Language				
English	6704 (91)	4933 (91)	1771 (91)	.011
Spanish	310 (4)	245 (5)	65 (3)	
Other/unknown	348 (5)	239 (4)	109 (6)	
Total	7362 (100)	5417 (100)	1945 (100)	
Insurance				
Private	3344 (45)	2489 (46)	855 (44)	< .001
Medicare	3428 (47)	2467 (46)	961 (49)	
Medicaid	362 (5)	272 (5)	90 (5)	
None	228 (3)	189 (3)	39 (2)	
Total	7362 (100)	5417 (100)	1945 (100)	
Marital status				
Married/partnered	3522 (48)	2417 (45)	1105 (57)	< .001
Single	1591 (22)	1166 (22)	425 (22)	
Widowed	979 (13)	865 (16)	114 (6)	
Divorced/separated	757 (10)	618 (11)	139 (7)	
Other/unknown	513 (7)	351 (6)	162 (8)	
Total	7362 (100)	5417 (100)	1945 (100)	
Comorbidities				
Chronic kidney disease (moderate/severe)	293 (4)	175 (3)	118 (6)	< .001
No chronic kidney disease (moderate/severe)	7069 (96)	5242 (97)	1827 (94)	
Total	7362 (100)	5417 (100)	1945 (100)	
Chronic obstructive pulmonary disease	1504 (20)	1169 (22)	335 (17)	< .001
No chronic obstructive pulmonary disease	5858 (80)	4248 (78)	1610 (83)	
Total	7362 (100)	5417 (100)	1945 (100)	
Congestive heart failure	694 (9)	439 (8)	255 (13)	< .001
No congestive heart failure	6668 (91)	4978 (92)	1690 (87)	
Total	7362 (100)	5417 (100)	1945 (100)	
Dementia	236 (3)	154 (3)	82 (4)	.0032
No dementia	7126 (97)	5263 (97)	1863 (96)	
Total	7362 (100)	5417 (100)	1945 (100)	
Diabetes mellitus	1223 (17)	815 (15)	408 (21)	< .001
No diabetes mellitus	6139 (83)	4602 (85)	1537 (79)	
Total	7362 (100)	5417 (100)	1945 (100)	
Glaucoma	249 (3)	197 (4)	52 (3)	.044
No glaucoma	7113 (97)	5220 (96)	1893 (97)	
Total	7362 (100)	5417 (100)	1945 (100)	

### Sources of care

The distribution of care included primary care/specialty co-management (for 25% of patients); primary care only (17%); urology only (13%); or another combination of specialty care (33%; Table 2). Men were more likely to get care from urologists and essentially did not receive care from gynecologists or urogynecologists.

**Table 2:** Patterns of Care by Sex

Characteristic	Overall (*n* = 7362)	Female (*n* = 5417)	Male (*n* = 1945)	*P* value
	No. (column %)			
Source of care				
Co-managed^*^	1828 (25)	1366 (25)	462 (24)	< .001
PCP only^†^	1288 (17)	1021 (19)	267 (14)	
Urologist only^‡^	938 (13)	374 (7)	564 (29)	
Gynecologist only^§^	195 (3)	195 (4)	0 (0)	
Urogynecologist only^║^	76 (1)	76 (1)	0 (0)	
Urologist, gynecologist, and/or urogynecologist only^¶^	637 (9)	633 (12)	4 (0)	
Other^#^	2400 (33)	1752 (32)	648 (33)	
Total	7362 (100)	5417 (100)	1945 (100)	
Initial medication				
Darifenacin	100 (1)	75 (1)	25 (1)	.006
Fesoterodine	55 (1)	41 (1)	14 (1)	
Flavoxate	9 (0)	9 (0)	0 (0)	
Hyoscyamine	83 (1)	65 (1)	18 (1)	
Imipramine	133 (2)	103 (2)	30 (2)	
Mirabegron	27 (0)	20 (0)	7 (0)	
Oxybutynin	1545 (21)	1184 (22)	361 (19)	
Solifenacin	428 (6)	324 (6)	104 (5)	
Tolterodine	485 (7)	359 (7)	126 (6)	
Trospium	91 (1)	75 (1)	16 (1)	
None	4406 (60)	3162 (58)	1244 (64)	
Total	7362 (100)	5417 (100)	1945 (100)	
Treatment				
Biofeedback	10 (0)	9 (0)	1 (0)	.24
No biofeedback	7352 (100)	5408 (100)	1944 (100)	
Total	7362 (100)	5417 (100)	1945 (100)	
OnabotulinumtoxinA	171 (2)	131 (2)	40 (2)	.36
No onabotulinumtoxinA	7191 (98)	5286 (98)	1905 (98)	
Total	7362 (100)	5417 (100)	1945 (100)	
Sacral nerve stimulation	74 (1)	56 (1)	18 (1)	.68
No sacral nerve stimulation	7288 (99)	5361 (99)	1927 (99)	
Total	7362 (100)	5417 (100)	1945 (100)	
Bladder augmentation	0 (0)	0 (0)	0 (0)	NA
No bladder augmentation	7362 (100)	5417 (100)	1945 (100)	
Total	7362 (100)	5417 (100)	1945 (100)	

NA, not applicable; PCP, primary care physician.

^*^Patients with a PCP who also received care from a urologist, gynecologist, and/or urogynecologist

^†^Patients with a PCP who did not receive care from a urologist, gynecologist, or urogynecologist

^‡^Patients with a urologist who did not receive care from a PCP, gynecologist, or urogynecologist

^§^Patients with a gynecologist who did not receive care from a PCP, urologist, or urogynecologist

^║^Patients with a urogynecologist who did not receive care from a PCP, gynecologist, or urologist

^¶^Patients with a urologist, gynecologist, and/or urogynecologist (at least two of three) who did not receive care from a PCP

^#^Patients who did not receive care from a PCP, urologist, gynecologist, or urogynecologist but received care from other specialists

### Medication use, classes, and persistence

Only 40% of patients received an OAB-related medication in the 2 years of follow-up. The most commonly prescribed medications were oxybutynin, tolterodine, and solifenacin. Patients who were co-managed by PCPs and a specialist had the highest medication receipt rate (48%), followed by patients seen by urogynecologists (47%), urologists (45%), a combination of specialists (41%), PCPs only (38%), other specialties (34%), and gynecologists only (30%) (*p* < .001); Supplementary Table 5 and Supplementary Table 6). Among patients with contraindications for anticholinergic treatment, 54% (127/236) of those with dementia and 46% (115/249) of those with glaucoma received OAB medication.

Among patients who received medications, the median number of OAB-related medications received in the subsequent 2 years was two (interquartile range, two to three; range one to seven). Among patients who received medication, the average duration of prescribed medication was 1.5 months (95% confidence interval [CI] 1.4–1.6 months; range, < 1–24 months).

In multivariable modeling, independent predictors of receipt of an OAB medication were increasing age (odds ratio [OR] 1.4 for every 10 years; 95% CI, 1.4–1.5), female sex (OR, 1.6 compared with male; 95% CI, 1.4–1.8), other marital status (OR, 0.5 compared with married; 95% CI, 0.4–0.6), widowed (OR, 0.7 compared with married; 95% CI, 0.6–0.9), diabetes (OR, 1.3; 95% CI, 1.1–1.5), and certain sources of care compared with PCP-only care: PCP/specialist co-management (OR, 1.8; 95% CI, 1.5–2.0), multiple specialists (OR, 1.4; 95% CI, 1.2–1.8), urogynecology (OR, 1.9; 95% CI, 1.2–3.1), and urology (OR, 2.2; 95% CI, 1.8–2.6). Marginally significant predictors of OAB medication prescribing were Hispanic race/ethnicity (OR, 1.3 compared with whites; 95% CI, 1.0–1.7) and chronic obstructive pulmonary disease (OR, 1.1; 95% CI, 1.0–1.3).

### Other treatments

Few patients received other treatments of any type. Less than 1% of patients received biofeedback, 2% of patients received onabotulinumtoxinA, and 1% of patients received sacral nerve stimulation.

### Anticholinergic burden

Over the 2-year follow-up, patients who received OAB medications had significantly higher anticholinergic burden than patients who did not (anticholinergic TSDD, 125 versus 46; *P* < .001). An increasing number of OAB medications were associated with a linear increase in anticholinergic burden: for each OAB medication prescribed there was an increase in anticholinergic TSDD of 31 (95% CI, 26–36; *P* < .001 for the increasing trend in anticholinergic burden).

## Discussion

In this 2-year longitudinal study of patients identified using an EHR phenotype for OAB, only 40% of patients were prescribed an OAB medication. Of patients who received an OAB medication, the mean number of medications prescribed was two and the average duration of prescribing was only 1.5 months. Use of non-medication treatments, like onabotulinumtoxinA, was infrequent, perhaps as it only received US Food and Drug Administration approval for treatment of OAB in adults not responsive to anticholinergics in 2013.

Compared with our EHR phenotyping approach, studies that identified patients based on medication or diagnosis claims had slightly longer, but still short, treatment durations with high discontinuation rates. Studies using only medication claims – which, by design, could not measure the rate of medication prescribing – generally found longer durations of treatment, generally ranging from 2 to 6 months [8, 16–18]. One study relying on a single diagnosis code to define OAB patients had medication fill rates over the course of 1 year of 24% [7]. Another study using diagnosis codes had a same-visit, EHR medication prescribing rate of 17% [19]. A third study relying on both diagnosis codes and a medication prescription found that 92% of patients discontinued or switched their first anticholinergic medication over 24 months, with a mean time to discontinuation or switch of 5 months [9]. A systematic review of OAB medication use found that 43–83% of patients discontinue anticholinergics in the first 30 days; up to half of patients may have experienced “primary nonadherence,” in which patients never fill their first prescription [20, 21].

Failure to recognize, address, and treat OAB may leave patients at risk for complications, adverse effects, and with worse quality of life. Guidelines support lifestyle modifications as primary therapy but pharmacologic therapy may be underutilized. Unfortunately, OAB patients with incontinence who initiated treatment have been shown to have poorer outcomes and higher costs than patients without OAB [22]. OAB is under-recognized, undertreated, and undermanaged in primary care. Patients do not bring up OAB with their physicians, primary care clinicians do not screen for OAB, and patients are generally dissatisfied with treatments for OAB [2, 6, 23, 24]. In our cohort, fewer than 20% of OAB patients were managed in the PCP setting only and fewer than 30% were co-managed between a PCP and a specialist. Despite the existence of guidance for primary care treatment of patients with OAB [25], a minority of patients with OAB seek care, many delay seeking care, few patients are diagnosed, and PCPs may prematurely refer patients for specialty care [24, 26–28]. Guidelines for referral should include failure to respond to pharmacologic therapy, uncertain diagnosis, microscopic or gross hematuria, or suspicion of bladder carcinoma [6].

Despite likely inadequate treatment, an increasing concern about OAB medications is anticholinergic burden in patients with high cumulative anticholinergic exposure. Studies have found associations between anticholinergic medication and brain atrophy, brain hypometabolism, progression to cognitive impairment, and increased healthcare utilization [29, 30]. Gray and colleagues found that dementia risk increased as the anticholinergic TSDD over 10 years increased when categories of 1–90, 91–365, 366–1095, and over 1095 were used [5]. Bladder antimuscarinics accounted for 10.5% of TSDD burden. We found a TSDD difference of 79 over 2 years between patients who did and did not receive OAB medications. Clinicians may not be aware of the cognitive risks associated with anticholinergic medications for OAB [31].

Our analysis has limitations that should be considered. First, our novel method of identifying OAB patients remains documentation-dependent. In order to be entered into the cohort, patients had to have at least one coded variable for OAB and clinicians or coders had to enter additional codes or documentation that indicated symptoms of OAB. Patients with unrecognized symptoms would not have such codes or documentation. Second, our OAB EHR phenotype of course does not have perfect sensitivity or PPV. We would have compared the sensitivity and PPV of our OAB EHR phenotype to other large-scale methods of OAB identification. However, as of November 2019, a PubMed search revealed no articles that reported the sensitivity or PPV for identifying OAB using claims or electronic health records. A conference presentation found that 25 different OAB-related ICD-9 codes had associations with OAB medications ranging from 0 to 21% (mean, 4%) [32].

Third, our measures of medication use represent medication prescribing, not fills or actual drug taking. Fourth, our detection period was restricted to 2 years, and may have identified patients with OAB characteristics prior to the identification period or prior receipt of OAB medications. Fifth, we could not include OAB health-related quality of life or other patient-reported outcome measures. Sixth, we only had access to documentation within our health system; care provided and documented elsewhere was not included. Seventh, patients were dichotomously defined as either having or not having OAB. We do not have a good method for determining disease duration, severity, or subtype. OAB disease duration and severity could be important in identifying differences between patients cared for by PCPs alone versus patients who saw specialists. OAB severity and subtype (e.g., incontinence-associated) could be associated with different patterns of OAB medication use. Eighth, we did not collect information about related symptoms, like constipation, which might also be associated with differential OAB medication use. Finally, as our results are based upon our health system, the results presented here may not be generalizable to other systems.

Despite these limitations, strengths of this study include our novel method of identifying an EHR phenotype for OAB and the relatively large sample of OAB patients. EHR phenotypes have the potential to expand understanding about the prevalence and treatment of OAB.

## Conclusions

OAB is a common, morbid condition that is under-recognized. For patients identified using an OAB EHR phenotype in an integrated healthcare system, physicians prescribed medication to only 40% of patients over 2 years. Specialists were roughly twice as likely to prescribe medication to patients compared with PCPs. Even for patients who received medication, the average duration of medication use was only 1.5 months. Patients appear to have their symptoms inadequately addressed, as evidenced by the small number of patients who receive active treatment and the very short average duration of treatment. Treated patients who received OAB medications, predominantly antimuscarinics, had an increased anticholinergic burden.

## Supplementary information

**Additional file 1: Table S1.** 37 OAB Structured Variables. **Table S2.** Final EHR Phenotype Model Variables and Coefficients. **Table S3.** Problem List Entries and Diagnosis Codes for Comorbid Conditions. **Table S4.** CPT Codes for Procedures. **Table S5.** Medication Prescribing by Patient Characteristics. **Table S6.** Cohort Characteristics by Source of Care.

## Data Availability

The dataset supporting the conclusions of this article is not publically available as the data contain identifiable information from patients who were receiving routine clinical care. An extract may be available from the corresponding author on reasonable request.

## Abbreviations

BOW: Bag of words
EHR: Electronic health record
ICD-9: International Statistical Classification of Diseases and Related Health Problems
ICS: International Continence Society
LASSO: Least absolute shrinkage and selector operator
OAB: Overactive bladder
PCP: Primary care physician
PPV: Positive predictive value
TSDD: Total standardized daily dose
