# Supplementary material for: Overactive bladder in an integrated delivery system: a longitudinal cohort study
Source: BMC Health Serv Res. 2020 May 20;20:447. doi: 10.1186/s12913-020-05315-1 (PMC7238545; doi:10.1186/s12913-020-05315-1)
Supplement: Supplementary file 1 — Additional file 1: Table S1. 37 OAB Structured Variables. Table S2. Final EHR Phenotype Model Variables and Coefficients. Table S3. Problem List Entries and Diagnosis Codes for Comorbid Conditions. Table S4. CPT Codes for Procedures. Table S5. Medication Prescribing by Patient Characteristics. Table S6. Cohort Characteristics by Source of Care. [file 12913_2020_5315_MOESM1_ESM.docx]

**Supplementary Table 1. 37 OAB Structured Variables**

| ***Medications***   - Darifenacin* - Fesoterodine* - Flavoxate * - Hyoscyamine - Imipramine - Mirabegron* - Oxybutynin* - Solifenacin* - Tolterodine* - Trospium*   ***ICD-9 codes***   - 596.51: Hypertonicity of bladder* - 596.52: Low bladder compliance - 596.54: Neurogenic bladder NOS* - 596.55: Detrusor sphincter dyssynergia* - 596.59: Other functional disorder of bladder* - 788.2: Retention of urine - 788.3: Urinary incontinence - 788.4: Frequency of urination and polyuria - 788.63: Urgency of urination* - 788.69: Other abnormality of urination - 788.91: Functional urinary incontinence - 788.99: Other symptoms involving urinary system | ***Problems***   - Bladder dysfunction - Bladder incontinence - Bladder muscle dysfunction-overactive* - Female stress incontinence - Functional disorder of bladder* - Genuine stress incontinence - Hyperactive bladder* - Incomplete emptying of bladder - Increased frequency of urination - Mixed incontinence - Neurogenic bladder* - Nocturia - Urge incontinence of urine - Urgent desire to urinate* - Urinary incontinence* |
| --- | --- |

* Clinical variables “highly likely” to identify patients with overactive bladder. The remainder of the variables were determined to be “likely” to identify patients with overactive bladder.

**Supplementary Table 2. Final EHR Phenotype Model Variables and Coefficients**

| **Variable** | **Beta Coefficient** |
| --- | --- |
| Intercept | -1.503457 |
| Diagnoses |  |
| - Hypertonicity of bladder | 1.317306 |
| - Neurogenic bladder NOS | -0.169972 |
| - Urgency of urination | 0.471121 |
| - Frequency of urination and polyuria | -0.545542 |
| Problems |  |
| - Bladder muscle dysfunction – overactive | 1.516689 |
| - Neurogenic Bladder | 0.956968 |
| - Urge incontinence of urine | 0.529970 |
| - Incomplete emptying of bladder | -0.516755 |
| - Increased frequency of urination | 0.888801 |
| - Nocturia | 0.426798 |
| - Urinary Incontinence | 0.7809972 |
| Natural language processing (“bag-of-words”) representation | 0.296255 |

**Supplementary Table 3. Problem List Entries and Diagnosis Codes for Comorbid Conditions**

| Diagnosis | ICD-9 codes | Problem list entries^*^ |
| --- | --- | --- |
| Chronic kidney disease | 582; 582.1; 582.2; 582.4; 582.8; 582.81; 582.89; 582.9; 583; 583.1; 583.2; 583.4; 583.6; 583.7; 585; 586; 588; 588.1; 588.8; 588.9; 582.0; 583.0; 588.0 | Elevated creatinine, end stage renal disease, nephrectomy, nephritis, nephrotic syndrome, polycystic kidneys, proteinuria, chronic renal dysfunction, acute renal failure, renal insufficiency, glomerulonephritis, hydronephrosis, early renal disease, hemodialysis, peritoneal dialysis, nephropathy, kidney transplant, interstitial nephritis |
| Chronic obstructive pulmonary disorder | 490; 491; 491.1; 491.2; 491.21; 491.8; 491.9; 492; 492.8; 493; 493.01; 493.1; 493.11; 493.21;493.9; 493.91; 494; 495; 495.1; 495.2; 495.3; 495.4; 495.5; 495.6; 495.8; 495.9; 496; 500; 501; 502; 503; 504; 505; 506.4; 491.0; 491.20; 492.0; 493.0; 493.00; 493.10; 493.20; 493.90; 495.0 | Bronchiectasis, chronic obstructive pulmonary disease, pulmonary hypertension, restrictive pulmonary disease, chronic bronchitis, lung transplant, interstitial lung disease, bronchopulmonary dysplasia, asthma with status asthmaticus, asthma (acute exacerbation) |
| Congestive heart failure | 428; 428.0; 428.1; 428.9 | Congestive heart failure, impaired LV function |
| Dementia | 290; 290.0; 290.1; 290.10; 290.11; 290.12; 290.13; 290.2; 290.20; 290.21; 290.3; 290.4; 290.40; 290.41; 290.42; 290.43; 290.8; 290.9 | Dementia, Alzheimer's disease |
| Diabetes | 249.00; 249.01; 249.11; 249.20; 249.40; 249.41; 249.50; 249.60; 249.61; 249.70; 249.80; 249.81; 249.90; 249.91; 250.00; 250.01; 250.02; 250.03; 250.10; 250.11; 250.12; 250.13; 250.20; 250.21; 250.22; 250.23; 250.30; 250.31; 250.32; 250.33; 250.40; 250.41; 250.42; 250.43; 250.50; 250.51; 250.52; 250.53; 250.60; 250.61; 250.62; 250.63; 250.70; 250.71; 250.72; 250.73; 250.80; 250.81; 250.82; 250.83; 250.90; 250.91; 250.92; 250.93; 250; 250.0; 250.00; 250.01; 250.02; 250.03; 250.1; 250.10; 250.11; 250.12; 250.13; 250.2; 250.20; 250.21; 250.22; 250.23; 250.3; 250.30; 250.31; 250.32; 250.33; 250.4; 250.40; 250.41; 250.42; 250.43; 250.5; 250.50; 250.51; 250.52; 250.53; 250.6; 250.60; 250.61; 250.62; 250.63; 250.7; 250.70; 250.71; 250.72; 250.73 | Borderline diabetes mellitus, diabetes mellitus type 1, diabetes mellitus type 2, insulin infusion, intravenous insulin infusion |
| Glaucoma | 365.X | Glaucoma |

ICD-9, International Statistical Classification of Diseases and Related Health Problems-9; LV, left ventricular.
^*^At the time of this study, the electronic health record problem list at Partners HealthCare used a locally-developed coding scheme (ie, not a standardized terminology). Thus, we present the corresponding text entries.

**Supplementary Table 4. CPT Codes for Procedures**

| Procedure | CPT code |
| --- | --- |
| Biofeedback | 90875; 90876; 90901; 90911; E0746 |
| Bladder augmentation | 51960 |
| Botulinum toxin | 52287 |
| Sacral nerve stimulation | 64561; 64581; 64585; 64590; 64595; 95970; 95972; 95973 |

CPT, Current Procedural Terminology.

**Supplementary Table 5. Medication Prescribing by Patient Characteristics**

| Characteristic | Overall (n = 7,362) | OAB medication (n = 2,956) | No OAB medication (n = 4,406) | *P* value |
| --- | --- | --- | --- | --- |
|  | No. (row %) | | |  |
| Sex |  |  |  |  |
| Female | 5,417 (100) | 2,255 (42) | 3,162 (58) | < .001 |
| Male | 1,945 (100) | 701 (36) | 1,244 (64) |  |
| Total | 7,362 (100) | 2,956 (40) | 4,406 (60) |  |
| Age |  |  |  |  |
| Less than 65 years old | 3,646 (100) | 1,201 (33) | 2,445 (67) | < .001 |
| 65 years old or older | 3,716 (100) | 1,755 (47) | 1,961 (53) |  |
| Total | 7,362 (100) | 2,956 (40) | 4,406 (60) |  |
| Race/ethnicity |  |  |  |  |
| White/Caucasian | 6,103 (100) | 2,489 (41) | 3,614 (59) | < .001 |
| Black/African American | 338 (100) | 139 (41) | 199 (59) |  |
| Hispanic/Latino | 270 (100) | 113 (42) | 157 (58) |  |
| Asian/Pacific Islander | 125 (100) | 58 (46) | 67 (54) |  |
| Other/unknown | 526 (100) | 157 (30) | 369 (70) |  |
| Total | 7,362 (100) | 2,956 (40) | 4,406 (60) |  |
| Language |  |  |  |  |
| English | 6,704 (100) | 2,698 (40) | 4,006 (60) | .81 |
| Spanish | 310 (100) | 124 (40) | 186 (60) |  |
| Other | 348 (100) | 134 (39) | 214 (61) |  |
| Total | 7,362 (100) | 2,956 (40) | 4,406 (60) |  |
| Insurance |  |  |  |  |
| Private | 3,344 (100) | 1,163 (35) | 2,181 (65) | < .001 |
| Medicare | 3,428 (100) | 1,676 (49) | 1,752 (51) |  |
| Medicaid | 362 (100) | 110 (30) | 252 (70) |  |
| None | 228 (100) | 7 (3) | 221 (97) |  |
| Total | 7,362 (100) | 2,956 (40) | 4,406 (60) |  |
| Marital status |  |  |  |  |
| Married/partnered | 3,522 (100) | 1,466 (42) | 2,056 (58) | < .001 |
| Single | 1,591 (100) | 565 (36) | 1,026 (64) |  |
| Widowed | 979 (100) | 461 (47) | 518 (53) |  |
| Divorced/separated | 757 (100) | 338 (45) | 419 (55) |  |
| Other/unknown | 513 (100) | 126 (25) | 387 (75) |  |
| Total | 7,362 (100) | 2,956 (40) | 4,406 (60) |  |
| Comorbidities |  |  |  |  |
| Chronic kidney disease (moderate/severe) | 293 (100) | 153 (52) | 140 (48) | < .001 |
| No chronic kidney disease (moderate/severe) | 7,069 (100) | 2,803 (40) | 4,266 (60) |  |
| Total | 7,362 (100) | 2,956 (40) | 4,406 (60) |  |
|  |  |  |  |  |
| Chronic obstructive pulmonary disease | 1,504 (100) | 696 (46) | 808 (54) | < .001 |
| No chronic obstructive pulmonary disease | 5,858 (100) | 2,260 (39) | 3,598 (61) |  |
| Total | 7,362 (100) | 2,956 (40) | 4,406 (60) |  |
|  |  |  |  |  |
| Congestive heart failure | 694 (100) | 355 (51) | 339 (49) | < .001 |
| No congestive heart failure | 6,668 (100) | 2,601 (39) | 4,067 (61) |  |
| Total | 7,362 (100) | 2,956 (40) | 4,406 (60) |  |
|  |  |  |  |  |
| Dementia | 236 (100) | 127 (54) | 109 (46) | < .001 |
| No dementia | 7,126 (100) | 2,829 (40) | 4,297 (60) |  |
| Total | 7,362 (100) | 2,956 (40) | 4,406 (60) |  |
|  |  |  |  |  |
| Diabetes mellitus | 1,223 (100) | 612 (50) | 611 (50) | < .001 |
| No diabetes mellitus | 6,139 (100) | 2,344 (38) | 3,795 (62) |  |
| Total | 7,362 (100) | 2,956 (40) | 4,406 (60) |  |
|  |  |  |  |  |
| Glaucoma | 249 (100) | 115 (46) | 134 (54) | .048 |
| No glaucoma | 7,113 (100) | 2,841 (40) | 4,272 (60) |  |
| Total | 7,362 (100) | 2,956 (40) | 4,406 (60) |  |
|  |  |  |  |  |
| Source of care |  |  |  |  |
| Co-managed^*^ | 1,828 (100) | 873 (48) | 955 (52) | < .001 |
| PCP only^†^ | 1,288 (100) | 492 (38) | 796 (62) |  |
| Urologist only^‡^ | 938 (100) | 426 (45) | 512 (55) |  |
| Gynecologist only^§^ | 195 (100) | 59 (30) | 136 (70) |  |
| Urogynecologist only^║^ | 76 (100) | 36 (47) | 40 (53) |  |
| Urologist, gynecologist, and/or urogynecologist only^¶^ | 637 (100) | 259 (41) | 378 (59) |  |
| Other^#^ | 2,400 (100) | 811 (34) | 1,589 (66) |  |
| Total | 7,362 (100) | 2,956 (40) | 4,406 (60) |  |
|  |  |  |  |  |

OAB, overactive bladder; PCP, primary care physician.
^*^Patients with a PCP who also received care from a Partners urologist, gynecologist, and/or urogynecologist.
^†^Patients with a PCP who did not receive care from a Partners urologist, gynecologist, or urogynecologist.
^‡^Patients with a urologist who did not receive care from a Partners PCP, gynecologist, or urogynecologist.
^§^Patients with a gynecologist who did not receive care from a Partners PCP, urologist, or urogynecologist.
^║^Patients with a urogynecologist who did not receive care from a Partners PCP, gynecologist, or urologist.
^¶^Patients with a urologist, gynecologist, and/or urogynecologist (at least two of three) who did not receive care from a Partners PCP.
^#^Patients who did not receive care from a PCP, urologist, gynecologist, or urogynecologist but received care from other Partners specialists.

**Supplementary Table 6. Cohort Characteristics by Source of Care**

| Characteristic | Overall (n = 7,362) | Co-managed^*^ (n = 1,828) |  | PCP only^†^ (n = 1,288) | Urologist only^‡^ (n = 938) | Gynecologist only^§^ (n = 195) | Urogynecologist only^║^ (n = 76) | Urologist, gynecologist, and/or urogynecologist only^¶^ (n = 637) | Other^#^ (n = 2,400) |
| --- | --- | --- | --- | --- | --- | --- | --- | --- | --- |
|  |  | No. (column %) | | | | | | | |
| Sex |  |  |  |  |  |  |  |  |  |
| Female | 5,417 (74) | 1,366 (75) |  | 1,021 (79) | 374 (40) | 195 (100) | 76 (100) | 633 (99) | 1,752 (73) |
| Male | 1,945 (26) | 462 (25) |  | 267 (21) | 564 (60) | 0 (0) | 0 (0) | 4 (1) | 648 (27) |
| Total | 7,362 (100) | 1,828 (100) |  | 1,288 (100) | 938 (100) | 195 (100) | 76 (100) | 637 (100) | 2,400 (100) |
| Age |  |  |  |  |  |  |  |  |  |
| Less than 65 years old | 3,646 (50) | 967 (53) |  | 563 (44) | 553 (59) | 128 (66) | 51 (67) | 418 (66) | 966 (40) |
| 65 years old or older | 3,716 (50) | 861 (47) |  | 725 (56) | 385 (41) | 67 (34) | 25 (33) | 219 (34) | 1,434 (60) |
| Total | 7,362 (100) | 1,828 (100) |  | 1,288 (100) | 938 (100) | 195 (100) | 76 (100) | 637 (100) | 2,400 (100) |
| Race/ethnicity |  |  |  |  |  |  |  |  |  |
| White/Caucasian | 6,103 (83) | 1,535 (84) |  | 1,117 (87) | 756 (81) | 149 (76) | 54 (71) | 524 (82) | 1,968 (82) |
| Black/African American | 338 (5) | 115 (6) |  | 58 (5) | 50 (5) | 8 (4) | 3 (4) | 21 (3) | 83 (3) |
| Hispanic/Latino | 270 (4) | 72 (4) |  | 34 (3) | 43 (5) | 12 (6) | 12 (16) | 41 (6) | 56 (2) |
| Asian/Pacific Islander | 125 (2) | 38 (2) |  | 17 (1) | 26 (3) | 7 (4) | 1 (1) | 10 (2) | 26 (1) |
| Other/unknown | 526 (7) | 68 (4) |  | 62 (5) | 63 (7) | 19 (10) | 6 (8) | 41 (6) | 267 (11) |
| Total | 7,362 (100) | 1,828 (100) |  | 1,288 (100) | 938 (100) | 195 (100) | 76 (100) | 637 (100) | 2,400 (100) |
| Language |  |  |  |  |  |  |  |  |  |
| English | 6,704 (91) | 1,678 (92) |  | 1,176 (91) | 851 (91) | 173 (89) | 61 (80) | 574 (90) | 2,191 (91) |
| Spanish | 310 (4) | 75 (4) |  | 41 (3) | 44 (5) | 13 (7) | 13 (17) | 47 (7) | 77 (3) |
| Other | 348 (5) | 75 (4) |  | 71 (6) | 43 (5) | 9 (5) | 2 (3) | 16 (3) | 132 (6) |
| Total | 7,362 (100) | 1,828 (100) |  | 1,288 (100) | 938 (100) | 195 (100) | 76 (100) | 637 (100) | 2,400 (100) |
|  |  |  |  |  |  |  |  |  |  |
| Insurance |  |  |  |  |  |  |  |  |  |
| Private | 3,344 (45) | 856 (47) |  | 506 (39) | 484 (52) | 108 (55) | 47 (62) | 377 (59) | 966 (40) |
| Medicare | 3,428 (47) | 874 (48) |  | 659 (51) | 411 (44) | 68 (35) | 23 (30) | 225 (35) | 1,168 (49) |
| Medicaid | 362 (5) | 80 (4) |  | 54 (4) | 42 (4) | 17 (9) | 6 (8) | 31 (5) | 132 (6) |
| None | 228 (3) | 18 (1) |  | 69 (5) | 1 (0) | 2 (1) | 0 (0) | 4 (1) | 134 (6) |
| Total | 7,362 (100) | 1,828 (100) |  | 1,288 (100) | 938 (100) | 195 (100) | 76 (100) | 637 (100) | 2,400 (100) |
| Marital status |  |  |  |  |  |  |  |  |  |
| Married/partnered | 3,522 (48) | 929 (51) |  | 524 (41) | 518 (55) | 103 (53) | 38 (50) | 354 (56) | 1,056 (44) |
| Single | 1,591 (22) | 422 (23) |  | 295 (23) | 237 (25) | 43 (22) | 19 (25) | 135 (21) | 440 (18) |
| Widowed | 979 (13) | 227 (12) |  | 272 (21) | 54 (6) | 16 (8) | 7 (9) | 56 (9) | 347 (14) |
| Divorced/separated | 757 (10) | 215 (12) |  | 150 (12) | 93 (10) | 16 (8) | 7 (9) | 64 (10) | 212 (9) |
| Other/unknown | 513 (7) | 35 (2) |  | 47 (4) | 36 (4) | 17 (9) | 5 (7) | 28 (4) | 345 (14) |
| Total | 7,362 (100) | 1,828 (100) |  | 1,288 (100) | 938 (100) | 195 (100) | 76 (100) | 637 (100) | 2,400 (100) |
| Comorbidities |  |  |  |  |  |  |  |  |  |
| Chronic kidney disease (moderate/severe) | 293 (4) | 115 (6) |  | 56 (4) | 40 (4) | 1 (1) | 0 (0) | 17 (3) | 64 (3) |
| No chronic kidney disease (moderate/severe) | 7,069 (96) | 1,713 (94) |  | 1,232 (96) | 898 (96) | 194 (99) | 76 (100) | 620 (97) | 2,336 (97) |
| Total | 7,362 (100) | 1,828 (100) |  | 1,288 (100) | 938 (100) | 195 (100) | 76 (100) | 637 (100) | 2,400 (100) |
|  |  |  |  |  |  |  |  |  |  |
| Chronic obstructive pulmonary disease | 1,504 (20) | 538 (29) |  | 369 (29) | 134 (14) | 35 (18) | 6 (8) | 92 (14) | 330 (14) |
| No chronic obstructive pulmonary disease | 5,858 (80) | 1,290 (71) |  | 919 (71) | 804 (86) | 160 (82) | 70 (92) | 545 (86) | 2,070 (86) |
| Total | 7,362 (100) | 1,828 (100) |  | 1,288 (100) | 938 (100) | 195 (100) | 76 (100) | 637 (100) | 2,400 (100) |
|  |  |  |  |  |  |  |  |  |  |
| Congestive heart failure | 694 (9) | 236 (13) |  | 185 (14) | 75 (8) | 10 (5) | 2 (3) | 18 (3) | 168 (7) |
| No congestive heart failure | 6,668 (91) | 1,592 (87) |  | 1,103 (86) | 863 (92) | 185 (95) | 74 (97) | 619 (97) | 2,232 (93) |
| Total | 7,362 (100) | 1,828 (100) |  | 1,288 (100) | 938 (100) | 195 (100) | 76 (100) | 637 (100) | 2,400 (100) |
|  |  |  |  |  |  |  |  |  |  |
| Dementia | 236 (3) | 78 (4) |  | 83 (6) | 13 (1) | 1 (1) | 2 (3) | 3 (0) | 56 (2) |
| No dementia | 7,126 (97) | 1,750 (96) |  | 1,205 (94) | 925 (99) | 194 (99) | 74 (97) | 634 (100) | 2,344 (98) |
| Total | 7,362 (100) | 1,828 (100) |  | 1,288 (100) | 938 (100) | 195 (100) | 76 (100) | 637 (100) | 2,400 (100) |
|  |  |  |  |  |  |  |  |  |  |
| Diabetes mellitus | 1,223 (17) | 385 (21) |  | 312 (24) | 148 (16) | 18 (9) | 9 (12) | 62 (10) | 289 (12) |
| No diabetes mellitus | 6,139 (83) | 1,443 (79) |  | 976 (76) | 790 (84) | 177 (91) | 67 (88) | 575 (90) | 2,111 (88) |
| Total | 7,362 (100) | 1,828 (100) |  | 1,288 (100) | 938 (100) | 195 (100) | 76 (100) | 637 (100) | 2,400 (100) |
| Initial medication |  |  |  |  |  |  |  |  |  |
| Darifenacin | 100 (1) | 33 (2) |  | 12 (1) | 13 (1) | 0 (0) | 1 (1) | 6 (1) | 35 (1) |
| Fesoterodine | 55 (1) | 21 (1) |  | 3 (0) | 8 (1) | 0 (0) | 0 (0) | 5 (1) | 18 (1) |
| Flavoxate | 9 (0) | 1 (0) |  | 1 (0) | 0 (0) | 2 (1) | 0 (0) | 2 (0) | 3 (0) |
| Hyoscyamine | 83 (1) | 24 (1) |  | 12 (1) | 8 (1) | 2 (1) | 1 (1) | 9 (1) | 27 (1) |
| Imipramine | 133 (2) | 44 (2) |  | 19 (1) | 18 (2) | 1 (1) | 2 (3) | 12 (2) | 37 (2) |
| Mirabegron | 27 (0) | 5 (0) |  | 6 (0) | 6 (1) | 0 (0) | 0 (0) | 3 (0) | 7 (0) |
| Oxybutynin | 1,545 (21) | 429 (23) |  | 260 (20) | 232 (25) | 35 (18) | 23 (30) | 130 (20) | 436 (18) |
| Solifenacin | 428 (6) | 131 (7) |  | 79 (6) | 65 (7) | 9 (5) | 3 (4) | 36 (6) | 105 (4) |
| Tolterodine | 485 (7) | 152 (8) |  | 86 (7) | 66 (7) | 9 (5) | 6 (8) | 49 (8) | 117 (5) |
| Trospium | 91 (1) | 33 (2) |  | 14 (1) | 10 (1) | 1 (1) | 0 (0) | 7 (1) | 26 (1) |
| None | 4,406 (60) | 955 (52) |  | 796 (62) | 512 (55) | 136 (70) | 40 (53) | 378 (59) | 1,589 (66) |
| Total | 7,362 (100) | 1,828 (100) |  | 1,288 (100) | 938 (100) | 195 (100) | 76 (100) | 637 (100) | 2,400 (100) |
|  |  |  |  |  |  |  |  |  |  |

PCP, primary care physician.
^*^Patients with a Partners PCP who also received care from a Partners urologist, gynecologist, and/or urogynecologist.
^†^Patients with a Partners PCP who did not receive care from a Partners urologist, gynecologist, or urogynecologist.
^‡^Patients with a Partners urologist who did not receive care from a Partners PCP, gynecologist, or urogynecologist.
^§^Patients with a Partners gynecologist who did not receive care from a Partners PCP, urologist, or urogynecologist.
^║^Patients with a Partners urogynecologist who did not receive care from a Partners PCP, gynecologist, or urologist.
^¶^Patients with a Partners urologist, gynecologist, and/or urogynecologist (at least two of three) who did not receive care from a Partners PCP.
^#^Patients who did not receive care from a Partners PCP, urologist, gynecologist, or urogynecologist but received care from other Partners specialists.
